# Method of lines for analysis of plane wave scattering by periodic arrays of magnetically-biased graphene strips

**DOI:** 10.1038/s41598-021-86882-z

**Published:** 2021-04-07

**Authors:** Mehri Ziaee Bideskan, Keyvan Forooraghi, Zahra Atlasbaf

**Affiliations:** Department of Electrical and Computer Engineering, Tarbiat Modares University, Tehran, 14115-194 Iran

**Keywords:** Engineering, Electrical and electronic engineering

## Abstract

In this paper, efficient analysis of the plane wave scattering by periodic arrays of magnetically-biased graphene strips (PAMGS) is performed using the semi-numerical, semi-analytical method of lines (MoL). In MoL, all but one independent variable is discretized to reduce a system of partial differential equations to a system of ordinary differential equations. Since the solution in one coordinate direction is obtained analytically, this method is time effective with a fast convergence rate. In the case of a multi-layered PAMGS, the governing equations of the problem are discretized concerning periodic boundary conditions (PBCs) in the transverse direction. The reflection coefficient transformation approach is then used to obtain an analytical solution in the longitudinal direction. Here, magnetically-biased graphene strips are modeled as conductive strips with a tensor surface conductivity which is electromagnetically characterized with tensor graphene boundary condition (TGBC). The reflectance and transmittance of different multi-layered PAMGS are carefully obtained and compared with those of other methods reported in the literature. Very good accordance between the results is observed which confirms the accuracy and efficiency of the proposed method.

## Introduction

Graphene, as a 2D material with hexagonal honeycomb lattice, has remarkable mechanical, electrical, and thermal properties such as astonishing strength and stiffness, optical transparency, high electronic mobility, and tunable surface conductivity^1–4^. The tunability of its surface conductivity by an applying electric or magnetic field bias is the main advantage of graphene over metals, which leads to the design of tunable devices including tunable THz absorbers^5,6^, graphene frequency selective surface with tunable polarization rotation characteristics^7^, etc. Moreover, the magnetically-biased graphene has anisotropic surface conductivity and shows non-reciprocal gyrotropy at microwave and optical frequencies^8^, which makes it a good candidate for designing nonreciprocal components^9^ and achieving enhanced Faraday rotation^10^.

To model the electromagnetic characteristics of graphene various methods have been implemented numerically. The graphene has been characterized using a circuit model through the partial element equivalent circuit (PEEC) method by Coa et al.^11^. The discontinuous Galerkin time domain (DGTD) method for the analysis of magnetically-biased graphene is reported by Li et al.^12^. The finite-difference time-domain (FDTD) method^13^ and the MoL^14^ are also reported for modelling magnetized graphene. Besides, magnetically biased graphene sheets are analyzed analytically by Heydari et al.^15^.

Alternatively, graphene-based periodic structures (GPS) with fascinating properties like perfect absorption of light in a specific range of frequency^16,17^ take advantage of the unique electromagnetic properties of graphene. The GPSs with numerous applications including THz absorbers^18^, THz filter^19^, and graphene-based frequency selective surfaces^20^, to name a few, have been reported in the literature. As a result, a fast and accurate method for the efficient analysis of GPSs is in need.

The study of electromagnetic waves interaction with periodic arrays of graphene has attracted great attention to the researchers recently. Different numerical methods, including the method of moments (MoM)^21^, finite-difference time-domain (FDTD)^22^, integral equations (IE)^23^, and Fourier modal method (FMM)^24–26^ have been reported for the diffraction analysis of GPSs in the absence of magnetic field bias while less effort has been made in case of magnetically-biased GPSs^20,27^.

In this paper, the semi-numerical, semi-analytical MoL has been provided for the efficient analysis of the plane wave scattering by PAMGS. In this method, we use of PBCs to take the periodicity of the PAMGS into account. Hence, the fields and the derivatives are to be discretized with finite differences according to the PBCs. Besides, the constitutive parameters of the cross section as well as the surface conductivity of graphene strips are discretized. In those parts of the cross-section where there is no graphene, the conductivity value is zero. Hence, there is no need to introduce approximate boundary condition at the interface of two adjacent layers where graphene exists as done by Khoozani *et. al*^27^.

Moreover, unlike fully numerical methods, the solution of the problem in one coordinate direction is obtained analytically in MoL which means there is no need to use absorbing boundary condition (ABC) such as perfectly matched layers (PML) in this direction. Besides, the analytical nature of the method saves a lot of computing time compared to fully numerical methods. The reflection coefficient transformation approach is introduced to obtain an analytical solution in this direction. In this approach, the structure under the study is divided into homogeneous layers and the known reflection coefficient in the last layer is transformed through layers and interfaces to obtain the reflectance. Having known the reflection coefficient in each layer, the transmission coefficient is obtained by transforming the fields in the opposite direction.

The rest of the paper is organized as follows: First, we present a brief description of the graphene properties. Then, we continue with the basic formulation of the problem. In this section, the MoL is introduced concisely. Afterwards, the plane wave incidence on a PAMGS for the two special cases of normal and oblique incidence is investigated. The reflection and transmission coefficients of a multi-layered PAMGS are obtained in the next subsection. Next, the numerical results of different PAMGSs obtained using the proposed method are presented.

**Figure 1:**
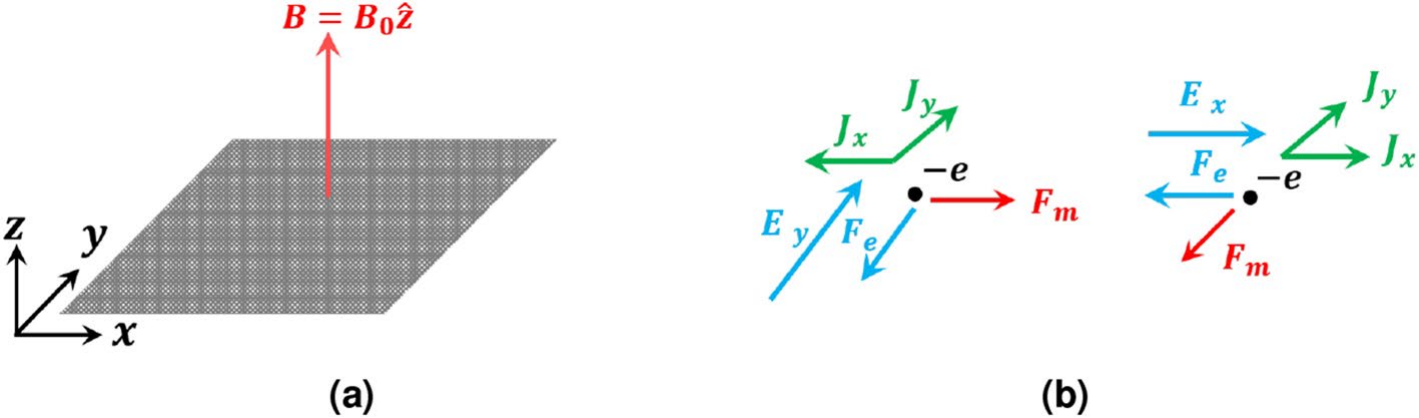
(**a**) Magnetically biased graphene sheet in *xy* plane with $B=B_0{\hat{z}}$, (**b**) Electron motion on the graphene plane in the presence of electric and magnetic fields.

## Graphene conductivity model

Consider a graphene sheet in the *xy* plane with a magnetic field bias $B=B_0{\hat{z}}$ as shown in Fig. 1a. As the result of its gapless electronic band structure and one-atom thickness, graphene can be treated as semiconductors^28,29^ characterizing with an anisotropic surface conductivity to be obtained through a quantum mechanical analysis using Kubo formulation^8,30,31^. However, in the case of magnetic field bias, there exists an excitonic energy gap which is opened due to the interaction of electrons with the magnetic field that can be significant at low temperature. At high temperature, the carrier density increases and consequently the screening effect enhances. Hence, the gap disappears and the graphene sheet’s electronic band structure remains gapless^32^.

The anisotropic nature of graphene’s conductivity can be interpreted by the electron motion in the *xy* plane under an electric field as shown in Fig. 1b^8^. For the case ${\mathbf{E} }=E_y{\hat{y}}$, the electric force $F_e$ will be in the $-{\hat{y}}$ direction. The acceleration of the electron in $-{\hat{y}}$ direction causes a Lorentz force $F_m$ in ${\hat{x}}$ direction. Consequently, the combination of these two forces results in two current components in $-{\hat{x}}$ and ${\hat{y}}$ directions. The case of ${\mathbf{E }}=E_x{\hat{x}}$ can be treated similarly in which the two current components are produced in ${\hat{x}}$ and ${\hat{y}}$ directions. Thus, the relation between the resultant surface currents and tangential electric fields is described as follows^8^

1 $$\begin{aligned} \left[ \begin{matrix} J_x \\ J_y \end{matrix}\right] =\left[ \bar{{\bar{\sigma }}}\right] . \left[ \begin{matrix} E_x \\ E_y \end{matrix}\right] ; \qquad \bar{{\bar{\sigma }}}\left( \omega , \mu _c, \tau , T, B_0\right) =\left[ \begin{matrix} \sigma _{xx}& -\sigma _{yx}\\ \sigma _{xy}& \sigma _{yy} \end{matrix}\right] . \end{aligned}$$

in which $\sigma _{xx}=\sigma _{yy}=\sigma _L$ and $\sigma _{xy}=-\sigma _{yx}=\sigma _T$ are the longitudinal (parallel to the electric field) and transversal (perpendicular to the electric field) components consisting of two parts modeling the intraband and interband electron transitions. However, in THz regime where $\hbar \omega <2 \mu _c$, the intraband transition is dominant^8,27^. Here, the optical properties of graphene are similar to those of Drude-type materials and as the result the quantum mechanical closed form expressions for $\sigma _L$ and $\sigma _T$ reduce to the classic Drude model as follows^8,27,33^

2 $$\begin{aligned} \begin{array}{l} \sigma _L=\sigma _0\displaystyle {\frac{1+j\omega \tau }{(\omega _c\tau )^2+(1+j\omega \tau )^2}},\\ \sigma _T=\sigma _0\displaystyle {\frac{\omega _c\tau }{(\omega _c\tau )^2+(1+j\omega \tau )^2}}, \end{array} \end{aligned}$$

with

3 $$\begin{aligned} \sigma _0=\frac{n_s e^2\tau v^2_F}{\mu _c}. \end{aligned}$$

where $\hbar$ is the reduced Plancks constant, *e* is the electron charge, $\mu _c$ is the chemical potential, $\tau$ is relaxation time, $\omega _c=eB_0v_F^2/\mu _c$ is the cyclotron frequency, $v_F=10^6 m/s$ is the Fermi velocity, $n_s$ is the carrier density, and *T* is the Kelvin temperature.

Alternatively, since the graphene sheet is modeled with a tensor *surface* conductivity, it can be electromagnetically treated as an special impedance boundary condition at the interface of adjacent layers called tensor graphene boundary condition (TGBC). The TGBCs are given by

4 $$\begin{aligned} \begin{array}{c} {\hat{n}}\times (\vec {E}_2-\vec {E}_1)=0,\\ {\hat{n}}\times (\vec {H}_2-\vec {H}_1)=[\bar{{\bar{\sigma }}}].\vec {E}_2. \end{array} \end{aligned}$$

in which ${\hat{n}}$ is the unit vector normal to the interface pointing from media 2 to media 1, $(\vec {E}_1, \vec {H}_1)$ and $(\vec {E}_2, \vec {H}_2)$ are the electric and magnetic fields in media 1 and 2, respectively.

These boundary conditions are then used in the next subsections to derive the reflection coefficient transformation formula through interfaces. Note that a time dependence of $e^{j\omega t}$ is assumed throughout this paper.

## Formulation of the problem

**Figure 2:**
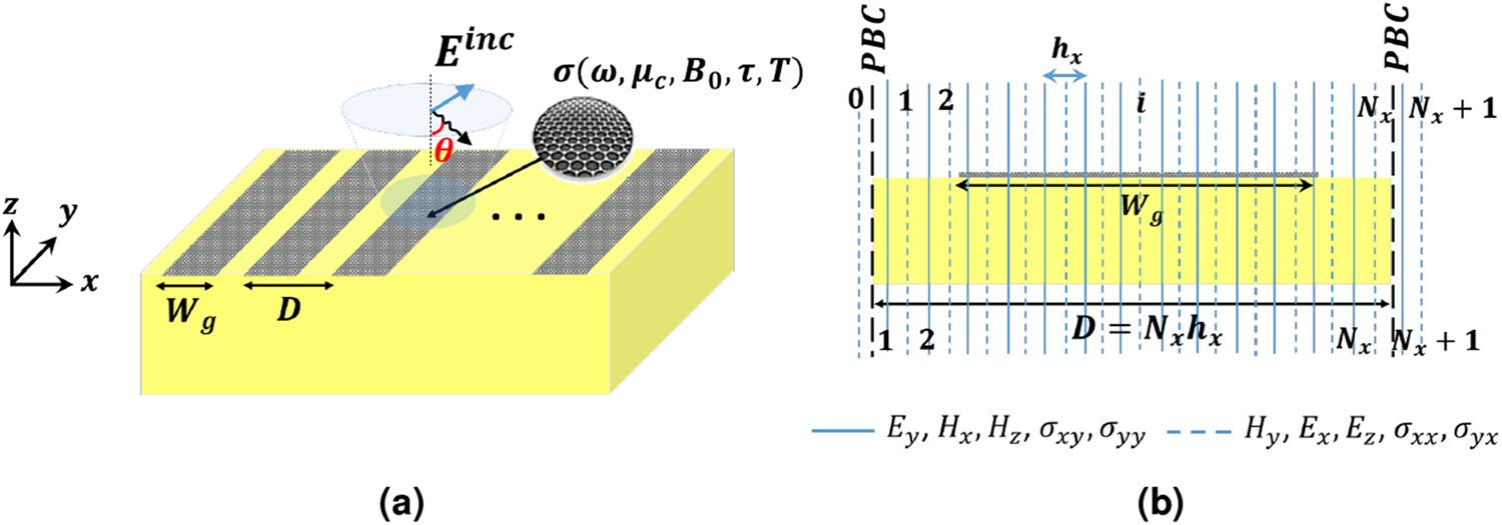
(**a**) Schematic of a PAMGS under illumination of an incident plane wave, (**b**) The discretized scheme of the PAMGS unit cell with periodic boundary conditions.

Consider a PAMGS with graphene strips of width $W_g$ and period length of *D* as shown in Fig. 2a in which the graphene strips are assumed to be infinitely long in the *y*-direction.

Despite graphene sheets with gapless electronic band structures, one-dimensional graphene strips with finite width have been proposed to obtain tunable band gaps where the energy gap is controlled by the strip width^28,29^. Besides, unlike infinite graphene sheets, graphene strips have non-uniform carrier density $n_s$ which means that the electrons near the edges of the strips experience different forces in compare to those in the middle of the strips. As the result, two different modes can be supported by the graphene strips namely edge modes and bulk modes with high localization near the edges and in the middle of the strips, respectively^33^. For chemically doped graphene, the net charge is zero. Here, the edge effects are confined to a strip width in order of the dopant’s Wigner-Seitz radius. Hence, for wide enough strips these edge effects can be neglected and the graphene strips can be modeled with the same conductivity parameters as those of an infinite sheet^33^.

### The method of lines

To start the analysis of a PAMGS and to consider its periodicity in *x*-direction, we normalize the electromagnetic fields with respect to the phase as follows:

5 $$\begin{aligned} \psi _i(x,y,z)=\psi _{0i}(x,y,z)e^{jk_xx}. \end{aligned}$$

in which $\psi _{0i}(x,y,z)$ is the non-normalized electromagnetic field component, $k_x$ is the propagation constant of the incident wave vector in *x*-direction and $\psi _i(x,y,z)$ is either $\textit{E}_i$ or $\textit{H}_i$ with $i=x, y, z$. These functions are periodic in the *x*-direction with period *D*.

Now we substitute Eq. (5) into Maxwell’s curl equations, which results in

6 $$\begin{aligned} -j\varepsilon _r\left[ \begin{matrix} E_y \\ E_x \end{matrix}\right] &= \displaystyle {\frac{\partial }{\partial {\bar{z}}}}\left[ \begin{matrix} -{\tilde{H}}_x \\ {\tilde{H}}_y \end{matrix}\right] -\left[ \begin{matrix} -D_{{\bar{x}}} \\ D_{{\bar{y}}} \end{matrix}\right] {\tilde{H}}_z,\nonumber \\ E_z& = -j\varepsilon _{r}^{-1}\left[ \begin{matrix} D_{{\bar{y}}}&D_{{\bar{x}}} \end{matrix}\right] \left[ \begin{matrix} -{\tilde{H}}_x \\ {\tilde{H}}_y \end{matrix}\right] . \end{aligned}$$

7 $$\begin{aligned} -j\left[ \begin{matrix} -{\tilde{H}}_x \\ {\tilde{H}}_y \end{matrix}\right]&= \displaystyle {\frac{\partial }{\partial {\bar{z}}}}\left[ \begin{matrix} E_y \\ E_x \end{matrix}\right] -\left[ \begin{matrix} D_{{\bar{y}}} \\ D_{{\bar{x}}} \end{matrix}\right] {\tilde{E}}_z,\nonumber \\ {\tilde{H}}_z&= j\left[ \begin{matrix} D_{{\bar{x}}}&- D_{{\bar{y}}} \end{matrix}\right] \left[ \begin{matrix} E_y \\ E_x \end{matrix}\right] . \end{aligned}$$

in which ${\bar{i}}=k_0i$ and ${\tilde{H}}_i=\eta _0H_i$ where $k_0=\omega \sqrt{\mu _0 \epsilon _0}$ and $\eta _0=\sqrt{\mu _0/\epsilon _0}$ are the free space wave number and wave impedance, respectively. Also, $D_{{\bar{y}}}=\partial /\partial {\bar{y}}$ and $D_{{\bar{x}}}=jk_{{\bar{x}}}+e^{jk_{{\bar{x}}}{\bar{x}}}\partial /\partial {\bar{x}}$ with $k_{{\bar{x}}}=k_x/k_0$.

Next, we eliminate the longitudinal field components $E_z$ and ${\tilde{H}}_z$ in Eqs. (6) and (7) to obtain generalized transmission line (GTL) equations as follows^34^

8 $$\begin{aligned} \displaystyle {\frac{d }{d {\bar{z}}}} \left[ \begin{matrix} E_y \\ E_x \end{matrix}\right]&= -j\left[ {R}_H\right] \left[ \begin{matrix} -{\tilde{H}}_x \\ {\tilde{H}}_y \end{matrix}\right] ,\nonumber \\ \left[ {R}_H\right]&= \left[ \begin{matrix} 1+{D}_{{\bar{y}}}{\varepsilon }_{r}^{-1}{D}_{{\bar{y}}}& {D}_{{\bar{y}}}{\varepsilon }_{r}^{-1}{D}_{{\bar{x}}}\\ {D}_{{\bar{x}}}{\varepsilon }_{r}^{-1}{D}_{{\bar{y}}} & 1+{D}_{{\bar{x}}}{\varepsilon }_{r}^{-1}{D}_{{\bar{x}}} \end{matrix}\right] . \end{aligned}$$

9 $$\begin{aligned} \displaystyle {\frac{d }{d {\bar{z}}}}\left[ \begin{matrix} -{\tilde{H}}_x\\ {\tilde{H}}_y \end{matrix}\right]&= -j\left[ {R}_E\right] \left[ \begin{matrix} E_y \\ E_x \end{matrix}\right] ,\nonumber \\ \left[ {R}_E\right]&= \left[ \begin{matrix} {\varepsilon }_{r}+{D}_{{\bar{x}}}{D}_{{\bar{x}}}& -{D}_{{\bar{x}}}{D}_{{\bar{y}}}\\ -{D}_{{\bar{y}}}{D}_{{\bar{x}}} &{\varepsilon }_{r}+{D}_{{\bar{y}}}{D}_{{\bar{y}}} \end{matrix}\right] . \end{aligned}$$

In this step, we discretize the fields and the derivatives in Eqs. (8) and (9) using finite differences. To this end, consider the unit cell of the PAMGS as shown in Fig. 2b with period length of *D* and $N_x$ discretization lines within the period. To start, the periodic functions of Eq. (5) are discretized as follows

10 $$\begin{aligned} e^{jk_{{\bar{x}}}{\bar{x}}}E_y={\mathbf{S} }_e{\mathbf{E }}_y; \quad e^{jk_{{\bar{x}}}{\bar{x}}}H_y={\mathbf{S }}_h{\mathbf{H} }_y. \end{aligned}$$

in which the ${\mathbf{S} }_e$ and ${\mathbf{S }}_h$ are diagonal matrices of size $N_x\times N_x$ whose elements are $e^{jk_{{\bar{x}}}ih_{{\bar{x}}}}$ and $e^{jk_{{\bar{x}}}(i+0.5)h_{{\bar{x}}}}$ for $i=1:N_x$, respectively. The discretized quantities are displayed in bold. Accordingly, the discretize field components of ${\mathbf{E }}_y$ and ${\mathbf{H }}_y$ are saved in column vectors of size $N_x\times 1$. Then, the phased normalized derivatives in the discretized domain are obtained as^34^

11 $$\begin{aligned} h_{{\bar{x}}}e^{jk_{{\bar{x}}}{\bar{x}}}\frac{\partial E_y}{\partial {{\bar{x}}}}={\mathbf{D }}_n{\mathbf{S }}_e{\mathbf{E} }_y; \qquad h_{{\bar{x}}}e^{jk_{{\bar{x}}}{\bar{x}}}\frac{\partial H_y}{\partial {{\bar{x}}}}=-{\mathbf{D} }^{*t}_n{\mathbf{S} }_h{\mathbf{H} }_y. \end{aligned}$$

with

12 $$\begin{aligned} {\mathbf{D} }_n= \begin{bmatrix} -s & s^*\\ & \ddots & \ddots & \\ & & \ddots & s^* \\ s^* & & & -s \end{bmatrix}. \end{aligned}$$

in which $s=e^{j\frac{k_xh_x}{2}}$. Hence, ${\mathbf{D} }_{{\bar{x}}}^e={\mathbf{D }}_{{\bar{x}}}{\mathbf{E }}_y$ and ${\mathbf{D} }_{{\bar{x}}}^h={\mathbf{D} }_{{\bar{x}}}{\mathbf{H }}_y$ are constructed according to Eq. (11).

Besides, since there is no variation in the *y*-direction we put ${\mathbf{D }}_{{\bar{y}}}=[0]_{N_x\times N_x}$ in Eqs. (8) and (9). As the result, the discretized GTL equations are obtained as follows:

13 $$\begin{aligned} {\frac{d }{d {\bar{z}}}} \left[ \begin{array}{cc} {\mathbf{E}} _y \\ {\mathbf{E }}_x \end{array}\right]&= -j \left[ {\mathbf{R }}_H\right] \left[ \begin{array}{cc} -\tilde{\mathbf{H }}_x \\ \tilde{\mathbf{H }}_y \end{array}\right] ,\nonumber \\ \left[ {R}_H\right]&= \varvec{\varepsilon }_{r}^{-1} \left[ \begin{array}{lc} \varvec{\varepsilon }_{r}{\mathbf{I} }& [0]\\ {[}0] & \varvec{\varepsilon }_{r}{\mathbf{I }}+{\mathbf{D }}_{{\bar{x}}}^e{\mathbf{D }}_{{\bar{x}}}^h \end{array}\right] . \end{aligned}$$

14 $$\begin{aligned} {\frac{d }{d {\bar{z}}}} \left[ \begin{array}{cc} -\tilde{\mathbf{H }}_x \\ \tilde{\mathbf{H }}_y \end{array}\right]&= -j\left[ {\mathbf{R }}_E\right] \left[ \begin{array}{cc} \mathbf{E }_y \\ \mathbf{E }_x \end{array}\right] ,\nonumber \\ \left[ {\mathbf{R }}_E\right]&= \left[ \begin{array}{cl} \varvec{\varepsilon }_{r}\mathbf{I } +{\mathbf{D }}_{{\bar{x}}}^h{\mathbf{D }}_{{\bar{x}}}^e & [0]\\ {[}0] & \varvec{\varepsilon }_{r}\mathbf{I } \end{array}\right] . \end{aligned}$$

where ${\mathbf{I} }$ is an identity matrix of $N_x$.

Using the definition ${\mathbf{E }}=[{\mathbf{E }}_y \quad {\mathbf{E }}_x]^t$ and ${\tilde{\mathbf{H }}}=[-\tilde{\mathbf{H }}_x \quad \tilde{\mathbf{H }}_y]^t$ where the superscript *t* refers to the matrix transpose, the solution to Eqs. (13) and (14) are given by^34^

15 $$\begin{aligned} {\mathbf{E }}(x,y,z)&= {\mathbf{T} }_E(x,y) (\bar{\mathbf{E }}_f(z)+\bar{\mathbf{E }}_b(z)), \end{aligned}$$

16 $$\begin{aligned} \tilde{\mathbf{H }}(x,y,z)&= \mathbf{T }_H(x,y) (\bar{\mathbf{H }}_f(z)+\bar{\mathbf{H }}_b(z)). \end{aligned}$$

in which ${\mathbf{T }}_E$ is a square matrix whose columns are the eigenvectors of matrix ${\mathbf{Q }}_E={\mathbf{R }}_E{\mathbf{R} }_H$. The corresponding eigenvalues of ${\mathbf{Q} }_E$ form the diagonal matrix of $\varvec{\Gamma ^2}$. Furthermore, it is proved that^34^

17 $$\begin{aligned} {\mathbf{T }}_H={\mathbf{R }}_E{\mathbf{T }}_E\varvec{\beta }^{-1}. \end{aligned}$$

where $\varvec{\beta }^2=\varvec{-\Gamma }^2$. Besides, $(\bar{{\mathbf{E }}}_f,\bar{{\mathbf{E} }}_b)$ and $(\bar{{\mathbf{H }}}_f,\bar{{\mathbf{H }}}_b)$ are forward and backward amplitudes of propagating electric and magnetic fields eigenmodes, respectively.

#### Plane wave incidence

According to Maxwell’s curl equations $\bigtriangledown \times \vec {E}=-j\omega \mu _0 \vec {H}$ and $\bigtriangledown \times \vec {H}=j\omega \varepsilon _0 \vec {E}$, for a plane wave with $\vec {E}=\vec {E}_0e^{-j(k_x{\hat{x}}+k_z{\hat{z}})}$ and $\vec {H}=\vec {H}_0e^{-j(k_x{\hat{x}}+k_z{\hat{z}})}$, the relation between transverse components of the electric and magnetic fields is given by

18 $$\begin{aligned} \vec {H}_t&= \omega \varepsilon _0\left( \frac{k_z}{k_0^2}\right) .\left( {\hat{z}}\times \vec {E}_t\right) , \end{aligned}$$

19 $$\begin{aligned} \vec {E}_t&= \omega \mu _0\left( \frac{k_z}{k_0^2}\right) .\left( \vec {H}_t\times {\hat{z}}\right) . \end{aligned}$$

where the subscript *t* refers to transverse components. In what follows, we investigate two special cases of normal and oblique incidence on a PAMGS using MoL.

**Figure 3:**
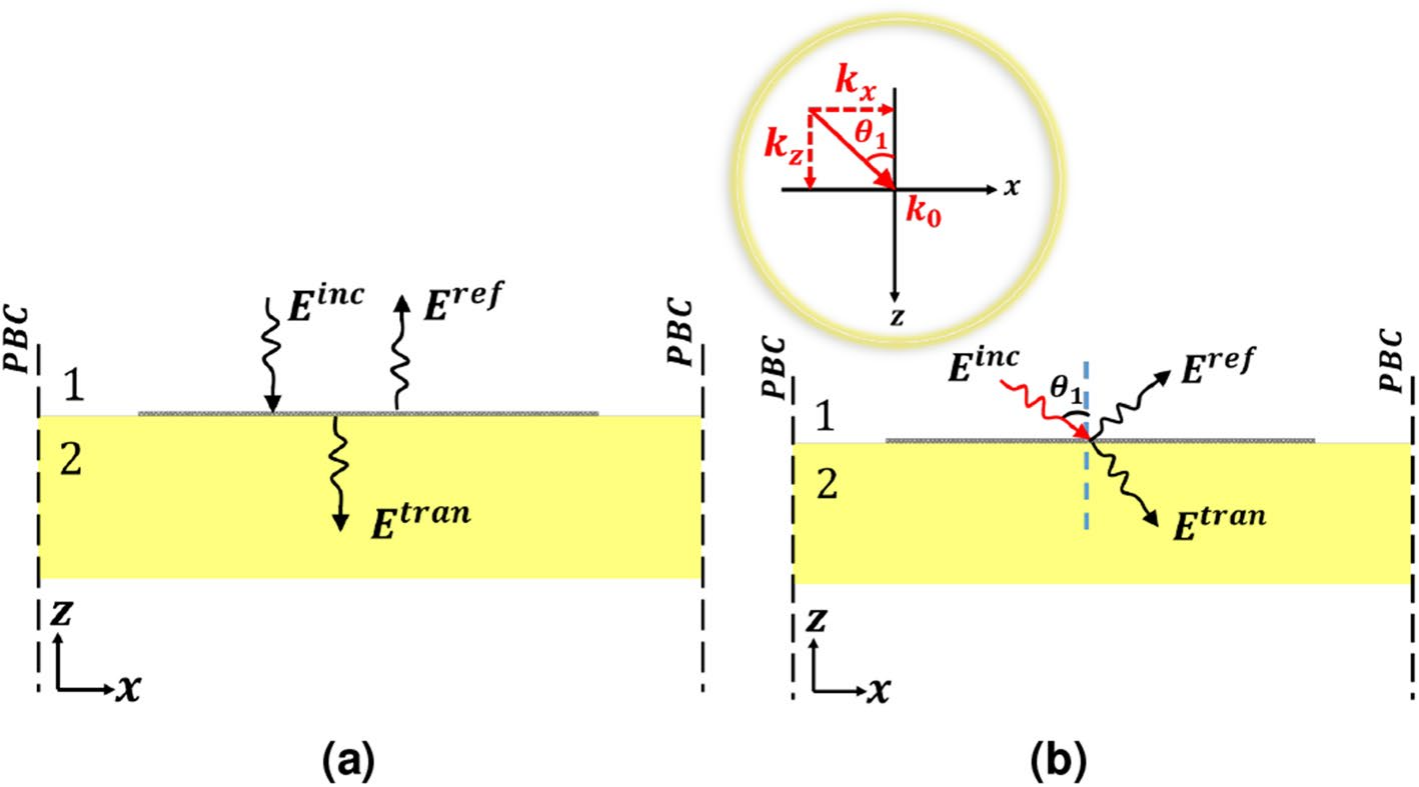
Plane wave incidence (**a**) Normal incidence (**b**) Oblique incidence.

#### Normal incidence

According to Fig. 3a, for the case of normal incidence we put $k_x=0$ in Eqs. (13) and (14) and compute the corresponding ${\mathbf{T }}_E$, ${\mathbf{T }}_H$ and $\varvec{\Gamma }$ in the two half spaces. Next, we define incident, reflected and transmitted electric and magnetic fields as follows:

20 $$\begin{aligned} {\mathbf{E} }^{inc}&= {\mathbf{T} }_{E1}\bar{\mathbf{E }}_{f1}, \qquad {\mathbf{H }}^{inc}={\mathbf{T} }_{H1}\bar{\mathbf{H }}_{f1},\nonumber \\ {\mathbf{E} }^{ref}&= {\mathbf{T} }_{E1}\bar{\mathbf{E }}_{b1}, \qquad {\mathbf{H} }^{ref}={\mathbf{T }}_{H1}\bar{\mathbf{H }}_{b1},\nonumber \\ {\mathbf{E }}^{tran}&= {\mathbf{T }}_{E2}\bar{\mathbf{E }}_{f2}, \qquad {\mathbf{H} }^{tran}={\mathbf{T} }_{H2}\bar{\mathbf{H }}_{f2}. \end{aligned}$$

By definition

21 $$\begin{aligned} {\mathbf{r} }&= \displaystyle {\frac{\mathbf{E }^{ref}}{\mathbf{E }^{inc}}},\nonumber \\ {{\mathbf{t }}}&= \displaystyle {\frac{{\mathbf{E} }^{tran}}{{\mathbf{E }}^{inc}}}. \end{aligned}$$

To obtain the unknown reflection and transmission coefficients of the PAMGS, the TGBCs at the interface of layers 1 and 2 must be applied. The discretized form of Eq. (4) reads as

22 $$\begin{aligned} \left[ \begin{array}{c} {\mathbf{E} }_y \\ {\mathbf{E} }_x \end{array}\right] _2&= \left[ \begin{array}{c}{\mathbf{E} }_y \\ {\mathbf{E} }_x \end{array}\right] _1 =\left[ \begin{array}{c}{\mathbf{E} }_y\\ {\mathbf{E }}_x \end{array}\right] ,\nonumber \\ \left[ \begin{array}{c} -\tilde{{\mathbf{H }}}_x \\ \tilde{\mathbf{H }}_y \end{array}\right] _2&= \left[ \begin{array}{c} -\tilde{\mathbf{H }}_x \\ \tilde{\mathbf{H }}_y \end{array}\right] _1+\eta _0 \left[ \begin{array}{ccc} -{\sigma }_{yy}{\mathbf{A} } & -{\mathbf{M }}_1{\sigma }_{yx}\\ {\mathbf{M} }_2{\sigma }_{xy} & -{\sigma }_{xx}{\mathbf{A }}\end{array}\right] \left[ \begin{array}{c} {\mathbf{E}} _y \\ {\mathbf{E} }_x \end{array}\right] , \end{aligned}$$

where ${\mathbf{A} }$ is a matrix of size $N_x\times N_x$ defined by

23 $$\begin{aligned} {\mathbf{A }}= \begin{bmatrix} {\mathbf{Z }}_{Ng}\\ & {\mathbf{I }}_{g}\\ & & {\mathbf{Z} }_{Ng} \end{bmatrix}. \end{aligned}$$

in which ${\mathbf{I }}_g$ and ${\mathbf{Z} }_{Ng}$ are identity and zero matrices of size $N_g$ and $(0.5(N_x-N_g))$, respectively where $N_g$ is the number of discretization lines on graphene strip. Moreover, according to Fig. 2b, ${\mathbf{E} }_y$ and $\mathbf{E }_x$ are discretized in different positions hence adding or subtracting them directly to obtain $\tilde{\mathbf{H }}_x$ or $\tilde{\mathbf{H }}_y$ is not allowed. As the result, the interpolation matrices $\mathbf{M }_1$ and $\mathbf{M }_2$ are introduced^35^ to obtain the field components in the correct position. Using the same definition for $\mathbf{E }$ and $\tilde{\mathbf{H }}$ as those of Eqs. (15) and (16), we rewrite Eq. (22) as follows

24 $$\begin{aligned} \mathbf{E }_2&= \mathbf{E }_1=\mathbf{E },\nonumber \\ \tilde{\mathbf{H }}_2&= \tilde{\mathbf{H }}_1+\eta _0 [\varvec{\bar{{\bar{\sigma }}}}]\mathbf{E }. \end{aligned}$$

Substituting $\mathbf{E }_1=\mathbf{E }^{inc}+\mathbf{E }^{ref}$ and $\mathbf{E }_2=\mathbf{E }^{tran}$ as well as $\mathbf{H }_1=\mathbf{H }^{inc}+\mathbf{H }^{ref}$ and $\mathbf{H }_2=\mathbf{H }^{tran}$ according to Eq. (20) in Eq. (24) and considering that $\mathbf{E }^{ref}=\mathbf{r }\mathbf{E }^{inc}$, we have

25 $$\begin{aligned}&\mathbf{T }_{E1}(\mathbf{I }+\mathbf{r })\bar{\mathbf{E }}_{f1}=\mathbf{T }_{E2}\bar{\mathbf{E }}_{f2},\nonumber \\&\mathbf{T }_{H2}\bar{\mathbf{H }}_{f2}=\mathbf{T }_{H1}(\bar{\mathbf{H }}_{f1}+\bar{\mathbf{H }}_{b1}) +\eta _0[\varvec{\bar{{\bar{\sigma }}}}]\mathbf{T }_{E2}\bar{\mathbf{E }}_{f2}. \end{aligned}$$

Furthermore, according to Eq. (18) for the case of normal incidence where $k_z=k_0$ we have

26 $$\begin{aligned} \bar{\mathbf{H }}_{fi}&= \bar{\mathbf{E }}_{fi}, \nonumber \\ \bar{\mathbf{H }}_{bi}&= -\bar{\mathbf{E }}_{bi}. \end{aligned}$$

where $i=1,2$ and the forward and backward magnetic fields are normalized to free space wave impedance $\eta _0$. Substituting Eq. (26) into Eq. (25), the reflection and transmission coefficients are obtained as

27 $$\begin{aligned} \mathbf{r }&= \frac{\mathbf{T }_{H2}^{-1}\mathbf{T }_{H1}-\mathbf{T }_{E2}^{-1}\mathbf{T }_{E1} +\mathbf{T }_{H2}^{-1}\eta _0[\varvec{\bar{{\bar{\sigma }}}}]\mathbf{T }_{E1}}{\mathbf{T }_{H2}^{-1} \mathbf{T }_{H1}+\mathbf{T }_{E2}^{-1}\mathbf{T }_{E1}-\mathbf{T }_{H2}^{-1}\eta _0[\varvec{\bar{{\bar{\sigma }}}}]\mathbf{T }_{E1}}, \end{aligned}$$

28 $$\begin{aligned} {\mathbf{t }}&= (\mathbf{I }+\mathbf{r }). \end{aligned}$$

**Figure 4:**
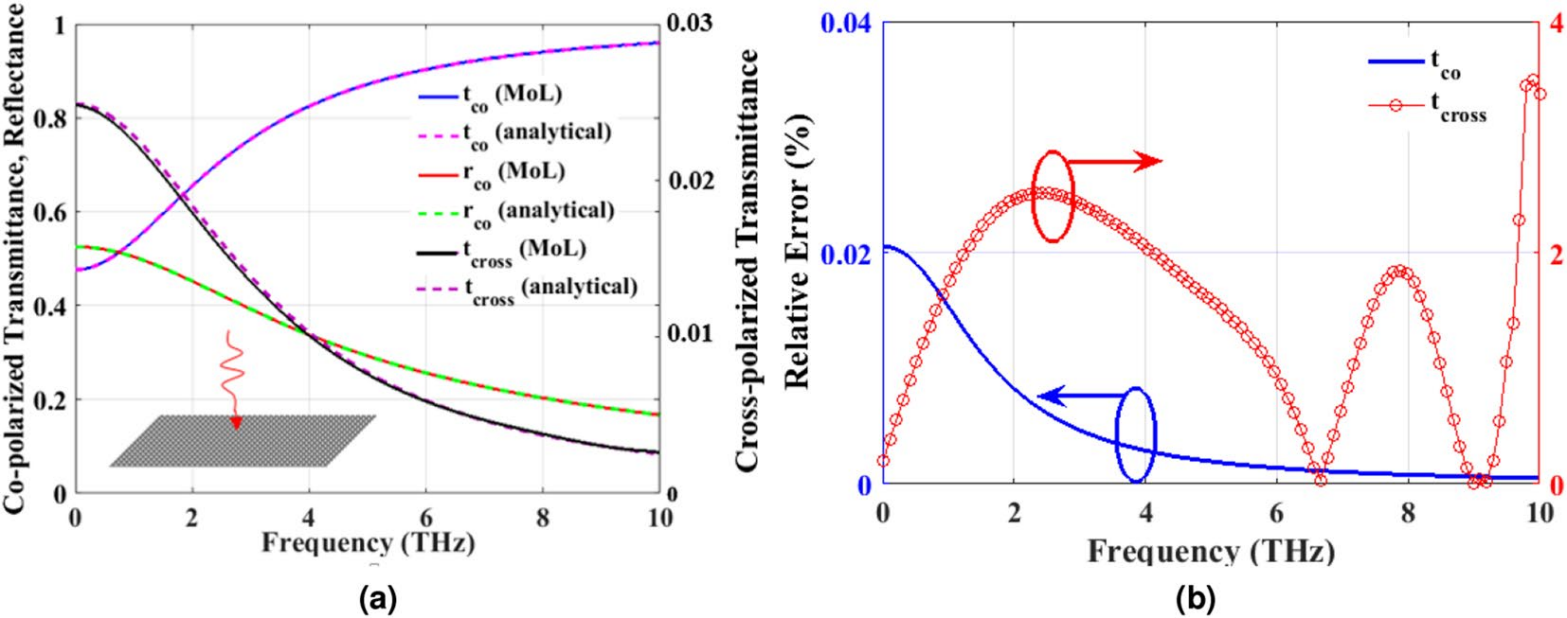
(**a**) Comparison of reflection and transmission coefficients of a free standing infinite graphene sheet obtained using both MoL (solid curves) and analytical formulation (dashed lines) for $\mu _c=0.5$ eV, $B_0=0.5$ T, $\tau =0.1$ ps and $T=300$ K, (**b**) Relative error between the MoL results and analytical formulations in the calculation of Co- and Cross-polarized transmission coefficients.

For $W_g=D$, the PAMGS shown in Fig. 3a reduces to an infinite graphene sheet at the interface of two infinite layers. In this case the reflection and transmission coefficients can be obtained analytically^8^. Figure 4a compares the obtained Co- and Cross-polarized reflection and transmission coefficients of a free standing infinite graphene sheet using MoL and analytical formulation. Besides, in order to examine the accuracy of the proposed method we calculated the relative error between our proposed method and those of analytical formulations for two cases of co- and cross-polarized transmittance as shown in Fig. 4b. The analytical results have been assumed as the reference in calculating this relative error. According to this figure, there is an excellent agreement between both sets of the results.

#### Oblique incidence

For the case of oblique incidence, the two particular cases of *TE*(*H*) and *TM*(*E*) polarized waves are investigated simultaneously. According to Fig. 3b, we put $k_z=k_0cos(\theta _i)$ with $i=1,2$ in Eqs. (18) and (19) which leads to

29 $$\begin{aligned} \bar{\mathbf{E }}_{fi}&= \left[ \mathbf{Z }\right] \bar{\mathbf{H }}_{fi},\nonumber \\ \bar{\mathbf{E }}_{bi}&= -\left[ \mathbf{Z }\right] \bar{\mathbf{H }}_{bi}. \end{aligned}$$

where

30 $$\begin{aligned} {[}\mathbf{Z }]=\left[ \begin{matrix} Z^{TE}_i\mathbf{I }&\\ &Z^{TM}_i\mathbf{I } \end{matrix}\right] . \end{aligned}$$

with

31 $$\begin{aligned} Z^{TE}_i=\frac{1}{cos(\theta _i)}, \qquad Z^{TM}_i=cos(\theta _i). \end{aligned}$$

Taking Eqs. (29) to (31) into account and following the same procedure as done in the previous case, the reflection and transmission coefficients with very small modification compared to normal incidence are obtained as follows

32 $$\begin{aligned} \mathbf{r }&= \frac{\mathbf{Z }_{21}\mathbf{T }_{H2}^{-1}\mathbf{T }_{H1}-\mathbf{T }_{E2}^{-1}\mathbf{T }_{E1} +\mathbf{Z }_2\mathbf{T }_{H2}^{-1}\eta _0[\bar{{\bar{\sigma }}}]\mathbf{T }_{E1}}{\mathbf{Z }_{21} \mathbf{T }_{H2}^{-1}\mathbf{T }_{H1}+\mathbf{T }_{E2}^{-1}\mathbf{T }_{E1}- \mathbf{Z }_2\mathbf{T }_{H2}^{-1}\eta _0[\bar{{\bar{\sigma }}}]\mathbf{T }_{E1}}, \end{aligned}$$

33 $$\begin{aligned} {\mathbf{t }}&= (\mathbf{I }+\mathbf{r }). \end{aligned}$$

where

34 $$\begin{aligned} \mathbf{Z }_{21}&= \left[ \begin{matrix} \displaystyle {\frac{Z_2^{TE}}{Z_1^{TE}}}\mathbf{I }&\\ &\displaystyle {\frac{Z_2^{TM}}{Z_1^{TM}}}\mathbf{I } \end{matrix}\right] , \end{aligned}$$

35 $$\begin{aligned} \mathbf{Z }_{2}&= \left[ \begin{matrix} Z_2^{TE}\mathbf{I }& \\ &Z_2^{TM}\mathbf{I } \end{matrix}\right] . \end{aligned}$$

**Figure 5:**
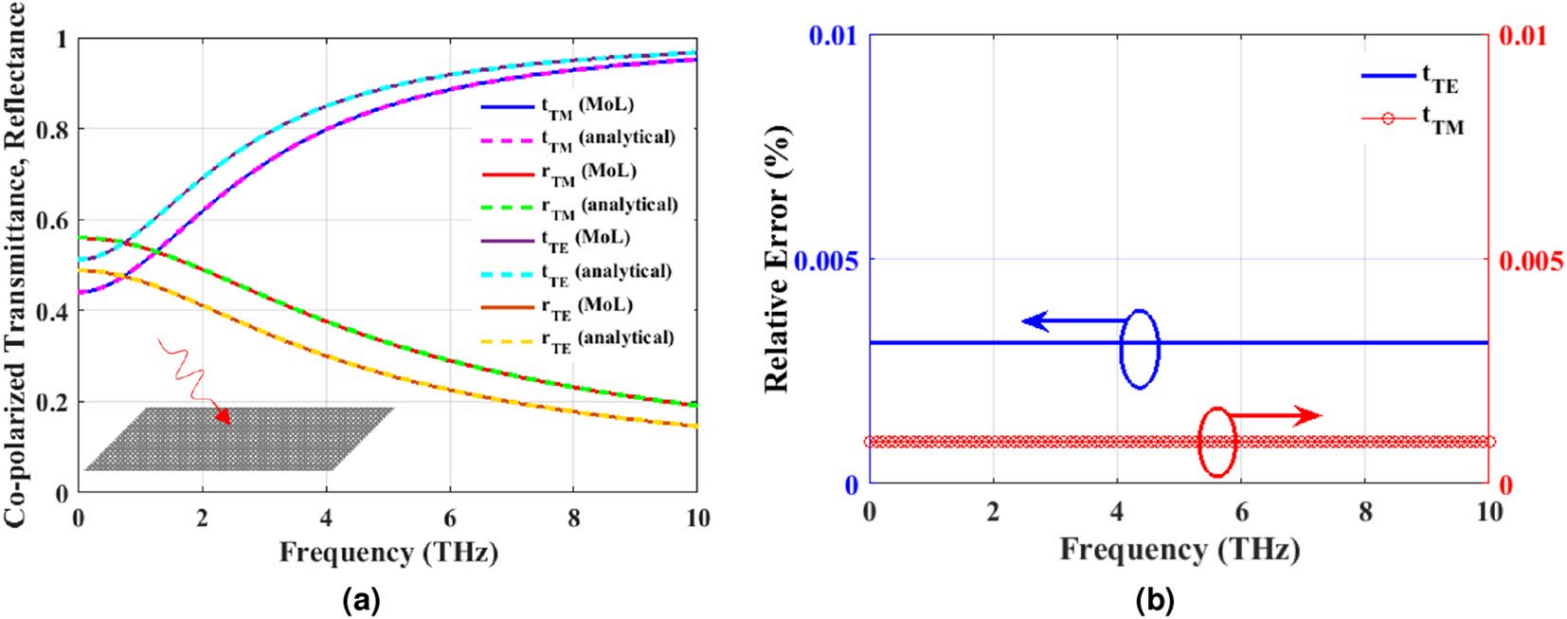
(**a**) Comparison of reflection and transmission coefficients of a free standing infinite graphene sheet obtained using both MoL (solid curves) and analytical formulation (dashed lines) for $\mu _c=0.5$ eV, $B_0=0.5$ T, $\tau =0.1$ ps and $T=300$ K at oblique incidence, (**b**) Relative error between the MoL results and analytical formulations in the calculation of TE and TM transmission coefficients.

It is worth mentioning that since the effect of the radiation angle $(\theta _1)$ on reflectance and transmittance is considered analytically through Eqs. (29) to (31), there is no need to recalculate the eigenvector matrices $(\mathbf{T }_{Ei}, \mathbf{T }_{Hi})$ in case of oblique incidence. The reflection and transmission coefficients of a free standing infinite graphene sheet under oblique incidence with $\theta _1=30^\circ$ are obtained using Eqs. (32) and (33) and compared with those of analytical solutions as shown in Fig. 5a. Again, we computed the relative error between our results and those of analytical formulations with analytical results being the reference as shown in Fig. 5b. This figure confirms the accuracy of our proposed method.

**Figure 6:**
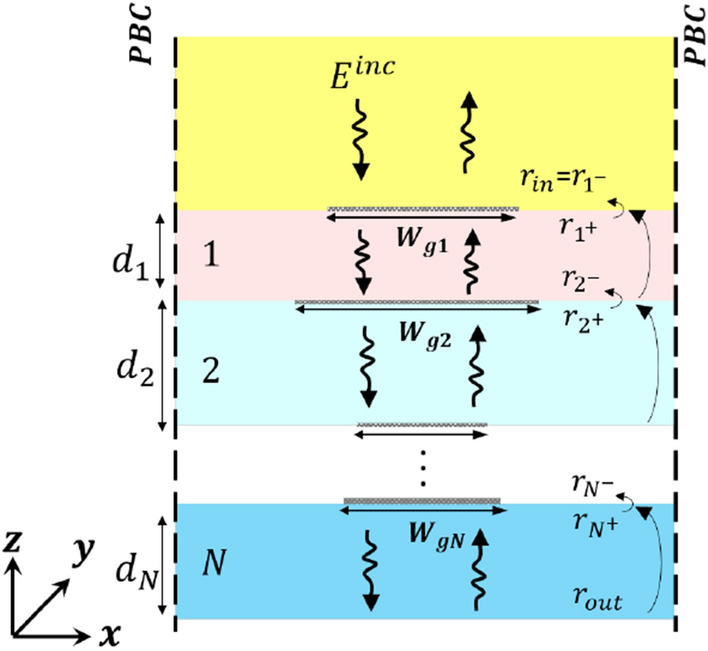
Reflection coefficient transformation through multilayer PAMGS. Generally, the graphene width in each layer can be different.

#### Multilayer PAMGS

In the analysis of a multilayer PAMGS as shown in Fig. 6, we use the reflection coefficient transformation approach to obtain the reflection and transmission coefficients of the whole periodic structure. In this approach the output reflection coefficient $(\mathbf{r }_{out})$ is assumed to be known and is to be transformed through layers and interfaces to obtain the input reflection coefficient $(\mathbf{r }_{in})$.

By definition

36 $$\begin{aligned} \mathbf{E }_{bi}=\mathbf{r }_i\mathbf{E }_{fi}. \end{aligned}$$

where *i* is the number of layer. On the other hand, $\bar{\mathbf{E }}_{f}(z)=e^{-\varvec{\Gamma }{\bar{z}}}\bar{\mathbf{E }}_{f}(0)$ and $\bar{\mathbf{E }}_b(z)=e^{\varvec{\Gamma }{\bar{z}}}\bar{\mathbf{E }}_b(0)$. Hence, according to Eq. (36) the reflection coefficient is transformed through the layers by

37 $$\begin{aligned} \mathbf{r }_{i^+}=e^{-\varvec{\Gamma _i}{\bar{d}}_i}\mathbf{r }_{(i+1)^-}e^{-\varvec{\Gamma _i}{\bar{d}}_i}. \end{aligned}$$

For $i=N$ we have $\mathbf{r }_{(i+1)^-}=\mathbf{r }_{out}$ which is set to zero in case of a semi-infinite layer. To calculate the reflection coefficient transformation formula through interfaces, the TGBCs have to be applied as follows

38 $$\begin{aligned}&\mathbf{T }_{Ei}(\mathbf{I }+\mathbf{r }_{i^+})\bar{\mathbf{E }}_{fi}=\mathbf{T }_{E(i-1)}(\mathbf{I } +\mathbf{r }_{i^-})\bar{\mathbf{E }}_{f(i-1)},\nonumber \\&\mathbf{T }_{Hi}(\mathbf{I }-\mathbf{r }_{i^+})\bar{\mathbf{H }}_{fi}=\mathbf{T }_{H(i-1)}(\mathbf{I } -\mathbf{r }_{i^-})\bar{\mathbf{H }}_{f(i-1)}+\eta _0[\varvec{\bar{{\bar{\sigma }}}}_i] \mathbf{T }_{Ei}(\mathbf{I }+\mathbf{r }_{i^+})\bar{\mathbf{E }}_{fi}. \end{aligned}$$

Now we substitute Eq. (29) into Eq. (38) and rearrange the formulas to obtain $\mathbf{r }_{i^-}$ as a function of $\mathbf{r }_{i^+}$ as follows

39 $$\begin{aligned} \mathbf{r }_{i^-}=([\mathbf{B }]-[\mathbf{C }]+[\mathbf{D }])([\mathbf{B }]+[\mathbf{C }]-[\mathbf{D }])^{-1}, \end{aligned}$$

with

40 $$\begin{aligned} {[}\mathbf{B }]&= (\mathbf{I }+\mathbf{r }_{i^+}),\nonumber \\ {[}\mathbf{C }]&= \mathbf{Z }_{(i-1)(i)}\mathbf{T }_{H(i-1)}^{-1}\mathbf{T }_{Ei}^{-1}\mathbf{T }_{E(i-1)}(\mathbf{I }-\mathbf{r }_{i^+}),\nonumber \\ {[}\mathbf{D }]&= \mathbf{Z }_{(i-1)}\mathbf{T }_{H(i-1)}^{-1}\eta _0[\bar{\bar{\sigma }}_i]\mathbf{T }_{E(i-1)}(\mathbf{I }+\mathbf{r }_{i^+}). \end{aligned}$$

Using the extracted recursive formulas for $i=N:1$, the reflection coefficient of the multilayer PAMGS as $\mathbf{r }_{in}=\mathbf{r }_{1^-}$ is obtained.

To obtain the transmission coefficient of the structure we should transform the fields in the opposite direction from the input to the output as follows

41 $$\begin{aligned} {\left\{ \begin{array}{ll} \mathbf{T }_{Ein}(\mathbf{I }+\mathbf{r }_{in})\bar{\mathbf{E }}_{fin}=\mathbf{T }_{E1}(\mathbf{I } +\mathbf{r }_{1^+})\bar{\mathbf{E }}_{f1},\\ \mathbf{T }_{E1}(e^{-\varvec{\Gamma }_1{\bar{d}}_1}+\mathbf{r }_{1^+}e^{\varvec{\Gamma }_1{\bar{d}}_1}) \bar{\mathbf{E }}_{f1}=\mathbf{T }_{E2}(\mathbf{I }+\mathbf{r }_{2^+})\bar{\mathbf{E }}_{f2}, \\ \mathbf{T }_{E2}(e^{-\varvec{\Gamma }_2{\bar{d}}_2}+\mathbf{r }_{2^+}e^{\varvec{\Gamma }_2{\bar{d}}_2}) \bar{\mathbf{E }}_{f2}=\mathbf{T }_{E3}(\mathbf{I }+\mathbf{r }_{3^+})\bar{\mathbf{E }}_{f3}, \\ ... \end{array}\right. } \end{aligned}$$

By definition

42 $$\begin{aligned} {\mathbf{t }}_{out}=\frac{\mathbf{T }_{EN}\bar{\mathbf{E }}_{fN}}{\mathbf{T }_{Ein}\bar{\mathbf{E }}_{fin}}. \end{aligned}$$

Hence,

43 $$\begin{aligned} {\mathbf{t }}_{out}=\prod _{i=1}^{N}\frac{(\mathbf{I }+\mathbf{r }_{in})}{(\mathbf{I } +\mathbf{r }_{1^+})}\frac{(e^{-\varvec{\Gamma }_i{\bar{d}}_i}+\mathbf{r }_{i^+}e^{\varvec{\Gamma }_i{\bar{d}}_i})}{(\mathbf{I }+\mathbf{r }_{(i+1)^+})}. \end{aligned}$$

Having known the reflection and transmission coefficients in each layer as well as $\bar{\mathbf{E }}_{fin}$ the complete solution of the structure is attainable.

**Figure 7:**
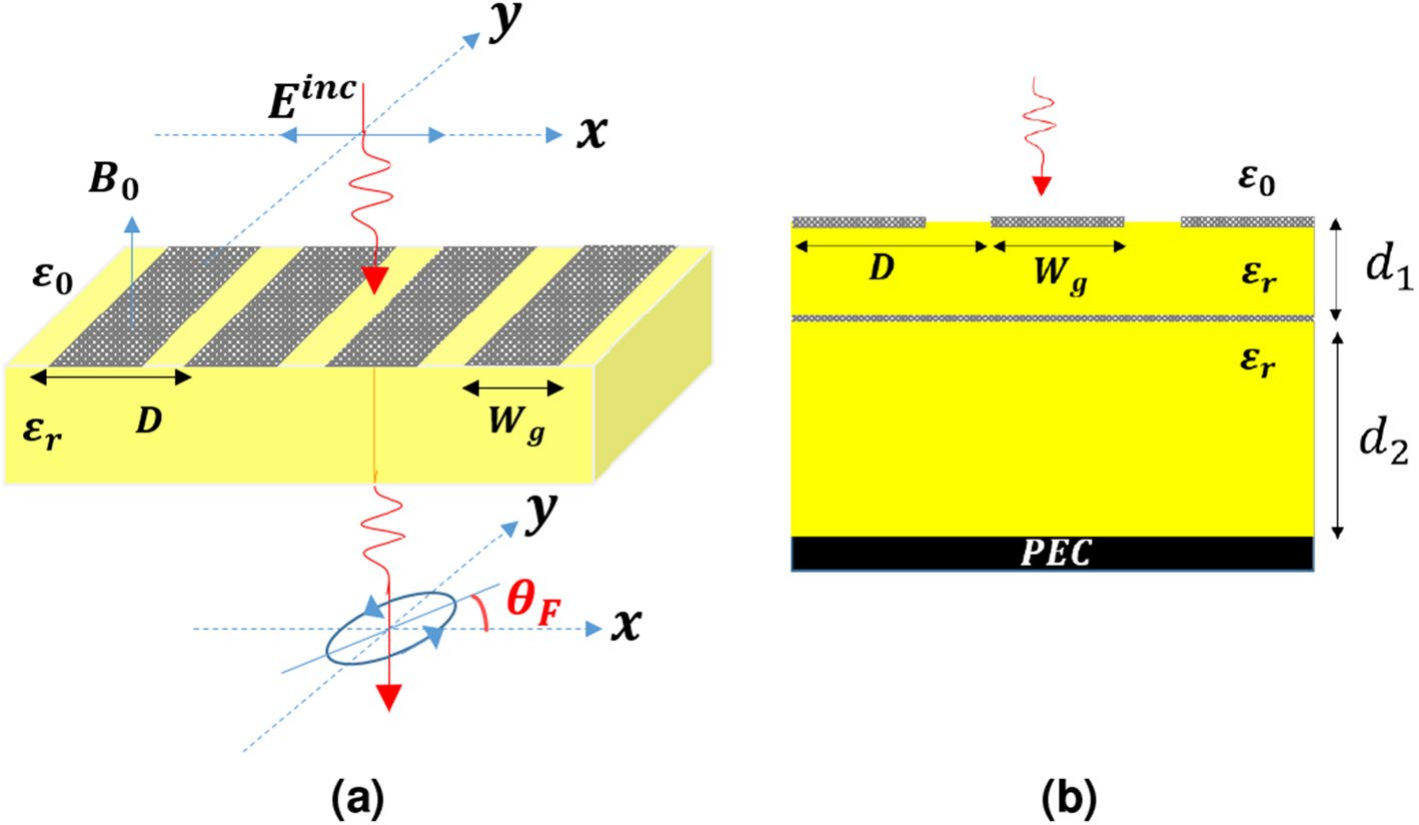
The schematic of (**a**) a PAMGS under illumination of *x*-directed wave at normal incidence. $\theta _F$ is the Faraday rotation angle, (**b**) The tunable THz absorber made of a multilayer PAMGS.

In short, we considered graphene as an especial impedance boundary condition at the interface of two adjacent layers due to its 2D structure and computed the reflection coefficient transformation formula through the interfaces, accordingly. Other 2D materials including Group-IV monoelemental honeycomb materials and binary compounds of group III-V elements can be treated similarly provided that we can model them as an appropriate boundary condition.

## Numerical results of different PAMGSs

To validate the proposed method, the numerical results for two different PAMGSs are obtained and compared with those of other methods. As the first example, the Faraday rotation effect achieved by a PAMGS as shown in Fig. 7a is investigated using MoL and compared with Fourier-Floquet plane wave expansion method^36^. Then, a multilayer PAMGS as a tunable THz absorber shown in Fig. 7b is analyzed. In this case, the obtained results are compared with those of the circuit model theory method. In both cases, the two sets of the results are in good accordance.

### Faraday rotation

The PAMGS shown in Fig. 7a is illuminated with a *x*-directed wave at normal incidence. In presence of a magnetic field bias, the direction of the transmitted electric field is deviated from that of the incident wave by angle $\theta _F$ called as Faraday rotation angle. By definition^36^

44 $$\begin{aligned} \theta _F=\tan ^{-1}\left( \frac{t_{cross}}{t_{co}}\right) , \end{aligned}$$

where $t_{cross}=t_{yx}$ and $t_{co}=t_{xx}$ are the transmission coefficients in *y*- and *x*-direction due to an *x*-directed incident wave, respectively.

On the other hand, the transmission efficiency is defined by^36^

45 $$\begin{aligned} T=\frac{n_2}{n_1}(t_{co}^2+t_{cross}^2). \end{aligned}$$

in which $n_1$ and $n_2$ are the refractive indices of incident and transmitted regions, respectively.

**Figure 8:**
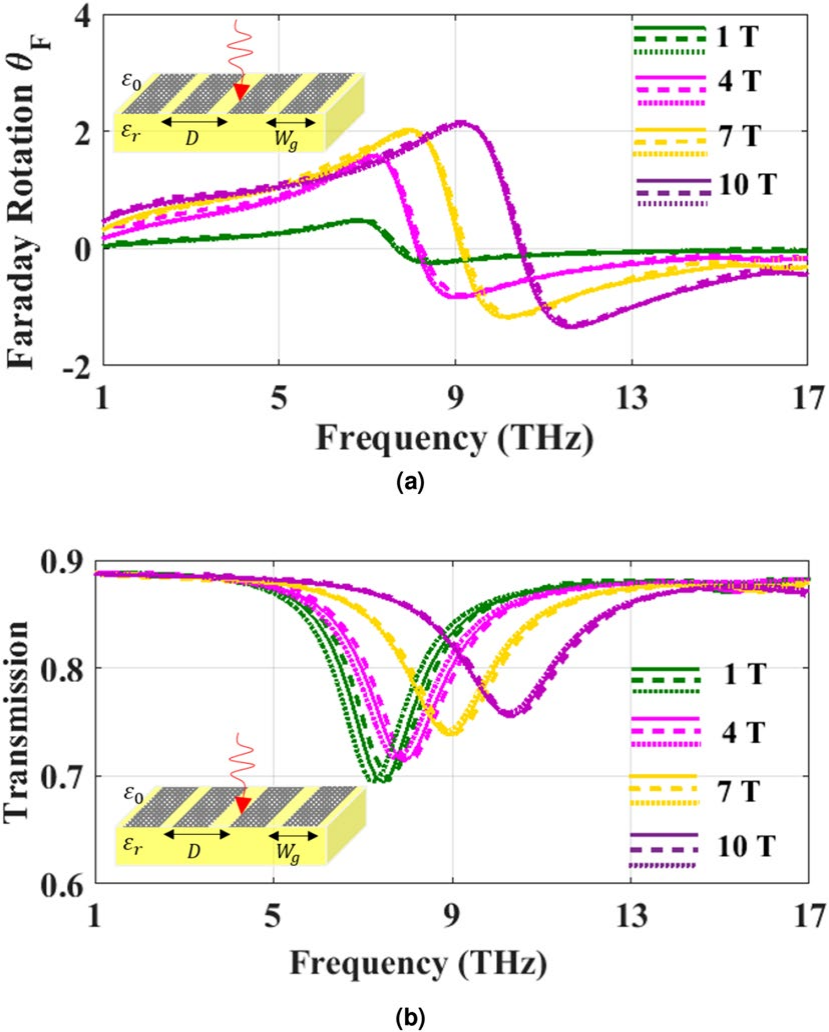
Comparison of (**a**) Faraday rotation angle and (**b**) transmission efficiency of the structure under study obtained using MoL (solid lines), those of Fourier-Floquet plane wave expansion method (dashed lines) and HFSS commercial software (dot lines) at different magnetic fields bias for $\mu _c=0.2$ eV, $\tau =0.1$ ps and $T=300$ K.

The Faraday rotation angle and the transmission efficiency of the structure shown in Fig. 7a with $D=2W_g=1 \mu m$ and $\varepsilon _r=4$ as a function of frequency are obtained using MoL and compared with those of Ref.^36^ and a commercial software as shown in Fig. 8a,b, respectively. In order to compare our numerical results with those obtained from commercial software, the structure shown in Fig. 7a is simulated using High Frequency Structural Simulator (HFSS) with its modal solution solver. It is worth mentioning that for this structure the number of discretization lines is set to $N_x=50$ for the MoL results to be converged. Moreover, to match the HFSS results to that of MoL, maximum Delta S is set to 0.0005 and took more than 40 minutes to run on an *Intel Core i7 8700K CPU, 32 GB RAM* desktop computer while the MoL took less than 4 seconds on the same computer. As it is seen the three sets of results agree very well. According to Fig. 8a,b the minimum transmittance occurs where the $\theta _F$ changes sign.

**Figure 9:**
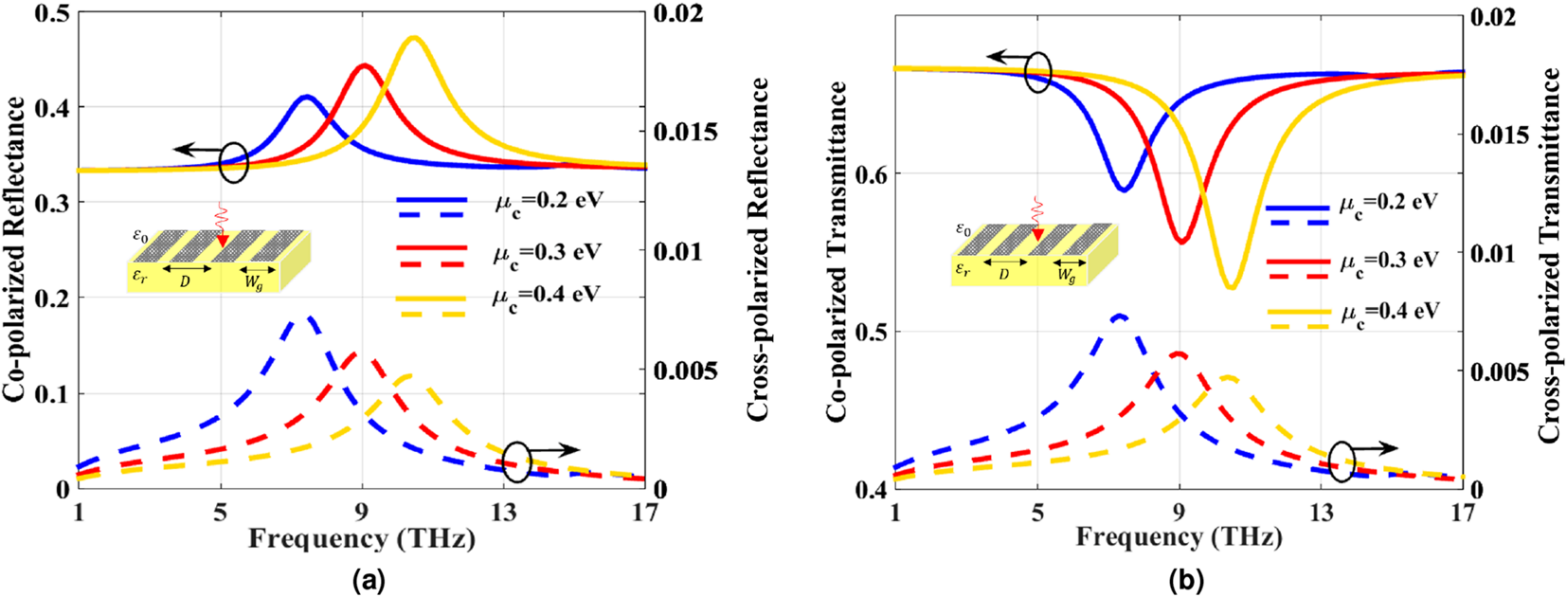
Co- and cross- (**a**) reflectance (**b**) and transmittance at different chemical potentials for $B_0=1$ T, $\tau =0.1$ ps and $T=300$ K.

Moreover, to investigate the effect of the chemical potential on reflectance and transmittance, the co- and cross-polarized reflectance and transmittance for different $\mu _c$ are obtained and shown in Fig. 9a,b, respectively. The resonance frequency of the PAMGS is blue-shifted as $\mu _c$ increases.

### Tunable THz absorber

The next example deals with a tunable THz absorber reported made of a multilayer PAMGS as shown in Fig.7b^6^. This absorber is composed of a perfect electric conductor (PEC), a continuous graphene sheet (CGS), and the periodic array of graphene strips (PAGS). The PEC, CGS and PAGS are separated with an intermediate dielectric layer. The PEC is used to suppress the transmission and increase the absorption.

**Figure 10:**
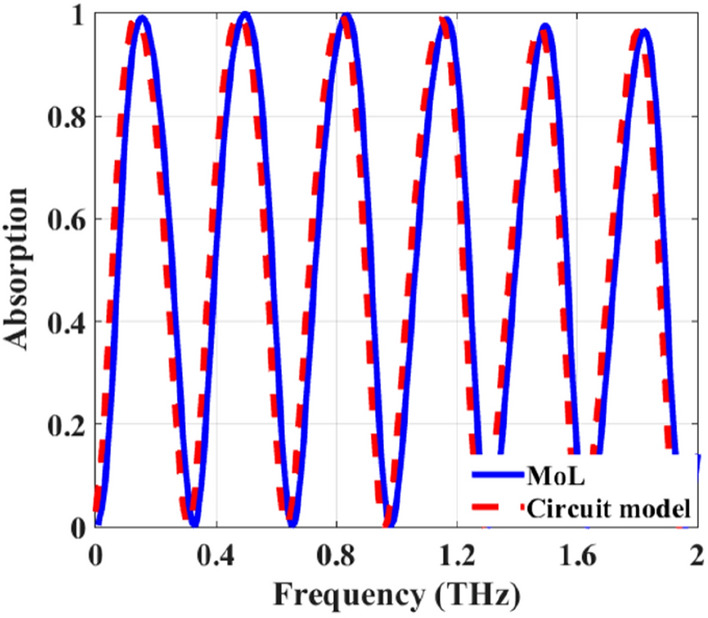
Absorption spectra of the tunable THz absorber of Fig.7b for $\mu _{c(CGS)}=0.25$ eV, $\mu _{c(PAGS)}=0.125$ eV, $\tau =0.1$ ps and $T=300$ K.

To evaluate the efficiency and accuracy of the proposed MoL in analyzing this absorber with $D=23.6 \mu m$, $W_g=18.28 \mu m$, $d_1=300 \mu m$, $d_2=0.3 \mu m$ and $\varepsilon _r=2.34$, the absorption of the structure as a function of frequency is obtained and compared with those of circuit model theory approach reported in literature^6^ as shown in Fig. 10. The graphene sheet and strips are assumed to be isotropic ($B_0=0$) and the structure is illuminated by an *x*-directed wave with normal incidence. As illustrated in Fig. 10, the obtained results using MoL agree very well with those of circuit model theory approach.

**Figure 11:**
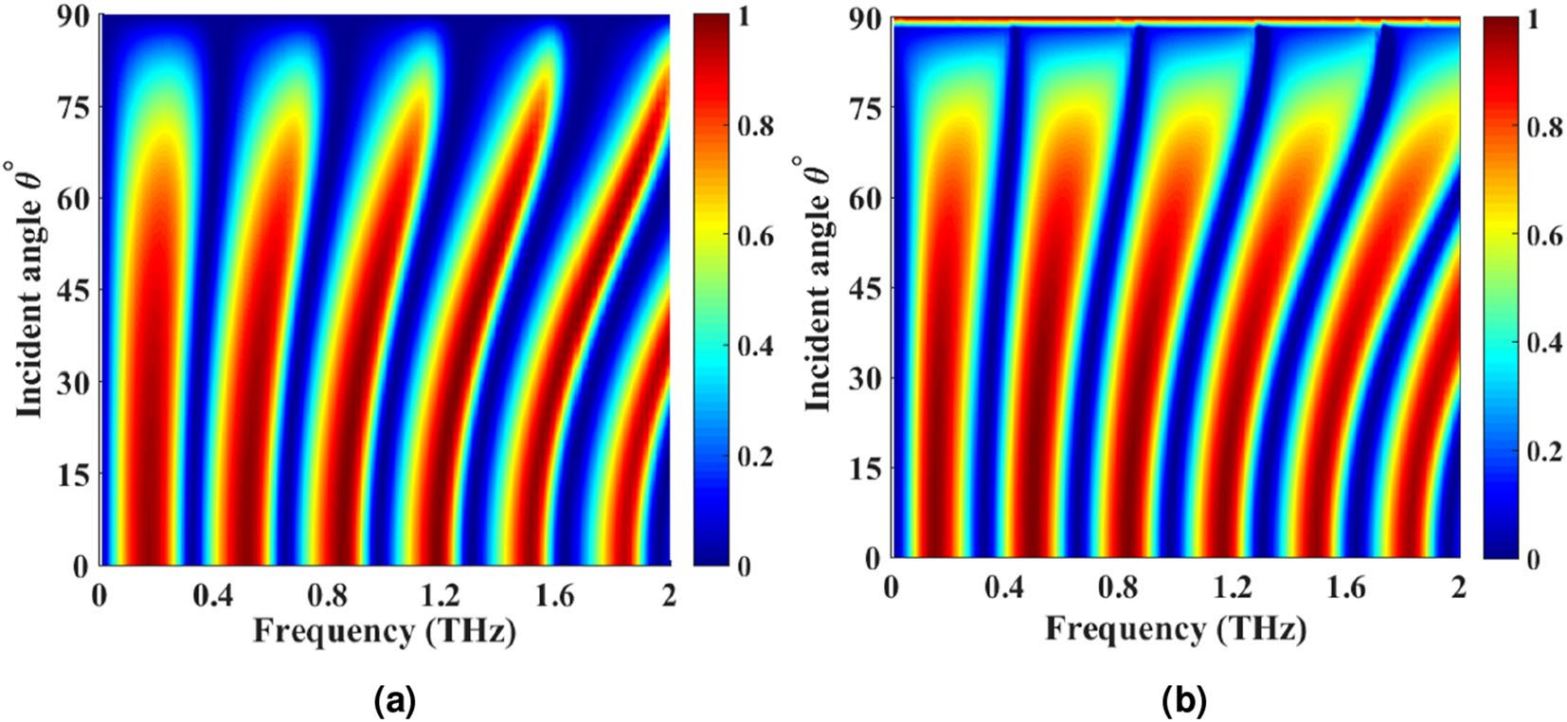
Absorption spectra of the tunable THz absorber of Fig.7b with oblique (**a**) *TM* and (**b**) *TE* polarized incident wave obtained by MoL for $\mu _{c(CGS)}=0.25$ eV, $\mu _{c(PAGS)}=0.125$ eV, $\tau =0.1$ ps and $T=300$ K.

Next, the effect of incident angle on absorption for both *TE* and *TM* polarizations is investigated. As shown in Fig. 11a,b, increasing the incident angle causes a blue shift in absorption peaks. Besides, the absorption of the structure for both *TE* and *TM* polarizations are almost the same. As the result, this absorber can be referred to as a polarization independent absorber^6^.

## Conclusion

In summary, we used the semi-numerical, semi-analytical method of lines (MoL) for the efficient analysis of the plane wave scattering in the most general case of oblique incidence for both *TE* and *TM* polarizations by multilayer periodic arrays of magnetically biased graphene strips (PAMGS). First, we discretized the governing equations in transverse directions concerning the periodic boundary condition using finite differences. Then, the reflection coefficient transformation approach was used to obtain the reflectance and transmittance of the multilayer structure analytically. Since the graphene parameters are discretized with finite differences in transverse directions, we set the surface conductivity to zero where graphene is absent. Hence, there is no need to introduce approximate boundary condition to model graphene. On the other hand, due to the semi-analytical nature of the method, there is no demand for the use of absorbing boundary conditions in the longitudinal direction unlike fully numerical methods. These advantages have made MoL a powerful computational tool for analyzing multi-layered graphene-based periodic structures. To validate the proposed method, numerical results for different PAMGSs are obtained using the proposed method and compared with those obtained by other methods reported in the literature. In all cases, the two sets of the results agree very well, which confirms the validity of the method. Analyzing 3D graphene-based periodic structures with 2D periodicity will be the subject of our future work.

## Data Availability

The datasets generated and analyzed during the current study are available from the corresponding author on reasonable request.
